# A charitable access program for patients with lysosomal storage disorders in underserved communities worldwide

**DOI:** 10.1186/s13023-020-01645-9

**Published:** 2021-01-06

**Authors:** Atul Mehta, Uma Ramaswami, Joseph Muenzer, Roberto Giugliani, Kurt Ullrich, Tanya Collin-Histed, Zoya Panahloo, Hartmann Wellhoefer, Joel Frader

**Affiliations:** 1 Department of Haematology, University College, London, UK; 2 University of North Carolina at Chapel Hill, Chapel Hill, NC USA; 3 Medical Genetics Service, HCPA, Department of Genetics, UFRGS, and INAGEMP, Porto Alegre, Brazil; 4 International Center for LSD, Hamburg, Germany; 5 International Gaucher Alliance, Dursley, UK; 6 Shire, a Takeda Company, Zurich, Switzerland; 7 Ann & Robert H. Lurie Children’s Hospital and Feinberg School of Medicine, Northwestern University, Chicago, IL USA

**Keywords:** Gaucher disease, Fabry disease, Hunter syndrome, MPS II, Access, Lysosomal storage disorders, Enzyme replacement therapy, Low/middle income economies, Humanitarian

## Abstract

**Background:**

Lysosomal storage disorders (LSDs) are rare genetic disorders, with heterogeneous clinical manifestations and severity. Treatment options, such as enzyme replacement therapy (ERT), substrate replacement therapy, and pharmacological chaperone therapy, are available for several LSDs, including Gaucher disease (GD), Fabry disease (FD), and Hunter syndrome (mucopolysaccharidosis type II [MPS II]). However, patients in some countries face challenges accessing treatments owing to limited availability of locally licensed, approved drugs.

**Methods:**

The Takeda LSD Charitable access program aims to meet the needs of individuals with GD, FD or MPS II with the greatest overall likelihood of benefit, in selected countries, through donation of ERT to nonprofit organizations, and support for medical capacity-building as well as family support via independent grants. Long-term aims of the program are to establish sustainable healthcare services delivered by local healthcare providers for patients with rare metabolic diseases. Patients receiving treatment through the program are monitored regularly, and their clinical data and progress are reviewed annually by an independent medical expert committee (MEC). The MEC also selects patients for enrollment completely independent from the sponsoring company.

**Results:**

As of 31 August, 2019, 199 patients from 13 countries were enrolled in the program; 142 with GD, 41 with MPS II, and 16 with FD. Physicians reported improvements in clinical condition for 147 (95%) of 155 patients with follow-up data at 1 year.

**Conclusions:**

The response rate for follow-up data at 1 year was high, with data collected for > 90% of patients who received ERT through the program showing clinical improvements in the majority of patients. These findings suggest that the program can benefit selected patients previously unable to access disease-specific treatments. Further innovative solutions and efforts are needed to address the challenges and unmet needs of patients with LSDs and other rare diseases around the world.

## Background

Lysosomal storage disorders (LSDs) are rare, complex conditions, the majority of which are caused by the intracellular accumulation of macromolecules due to the deficiency of a specific lysosome enzyme. These disorders are characterized by a broad range of clinical manifestations, depending on the specific substrate and the sites of accumulation, which vary according to the LSD type. They are typically progressive, and life-limiting and life-threatening in severe cases [1]. More than 60 different LSDs have been identified to date [2], with an estimated overall prevalence of 1 in 7000–8000 live births [3, 4].

Treatment options, including enzyme replacement therapy (ERT), substrate reduction therapy (SRT), and pharmacological chaperone therapy (PCT), are available for several LSDs, including Gaucher disease (GD), Fabry disease (FD), and Hunter syndrome (mucopolysaccharidosis type II [MPS II]). However, patients in some countries face challenges accessing treatments owing to low healthcare provider (HCP) awareness and education regarding rare diseases and the lack of locally licensed, approved, and reimbursed drugs, compounded by the high cost of enzyme replacement therapy, typically well beyond the healthcare budgets of developing countries. Further challenges include limited availability of local diagnostic services, including access to laboratory facilities and insufficient infrastructure. ERT treatments are typically intravenously administered recombinant proteins that require a complex supply chain, including temperature control.

For these 3 LSDs, published epidemiological data indicate a birth rate of ~ 1 in 40,000 individuals worldwide for GD [5]; FD has an incidence of ~ 1 in 40,000 males [6] and ~ 1 in 20,000 females [7], and MPS II has an incidence of ~ 1 in 93,000–200,000 males [8]. However, epidemiological data pertaining to rare disorders largely comes from countries with good access to diagnostic facilities and treatments and with high involvement in clinical studies and/or patient registries, predominantly across Europe, North America, Israel, Japan, and Australia [9]. Thus, a truly “global” representation of LSD epidemiology in less-developed regions, such as parts of Asia, Latin America, Africa, and the Middle East, is not available. Extrapolation of “global” prevalence and natural history data to underserved countries is problematic owing to potential regional variations in disease genotypes and phenotypes.

Charitable or humanitarian programs are run by several companies and non-governmental organizations (NGOs) in an attempt to address the needs of patients unable to access approved medicines. One such program is the LSD charitable access program, which was initiated by Shire (now a member of the Takeda group of companies) in 2012 with Project Hope, and with Direct Relief in 2014, in response to the unmet needs of patients with GD, FD, and MPS II in underserved communities. The program aims to take a holistic approach to treatment access, through the engagement of key non-company stakeholders, including patient advocacy organizations and external medical experts, with longer-term aims of establishing sustainable healthcare services provided by local clinicians and communities for patients with LSDs. This article describes the aims and structure of the LSD charitable access program and reports on patient outcomes for those receiving treatment through the program.

## Methods

### Overview of the Takeda LSD charitable access program

As part of the LSD charitable access program, Takeda donates ERTs for patients with GD, FD, and MPS II to nonprofit organizations and NGOs which, in turn, ship the drugs to HCPs in selected countries. The criteria for country selection are as follows: the products are not available due to lack of coverage or reimbursement by the local health care system; there are patients diagnosed with any of these 3 diseases; no local regulatory filing is planned by Takeda for the ERTs (or is more than 12–24 months away); and in-country capacity exists to safely administer the ERT intravenous infusions. Once a patient is enrolled in the program, ERT will be maintained life-long, provided the patient is benefiting from ERT, or until the therapy can be provided by the local healthcare system and reimbursement is possible. In addition, the program supports capacity-building efforts mainly via independent grants, which can make a significant contribution to local diagnostic services, disease awareness, as well as practical disease management educational programs for (rare) metabolic diseases, extending beyond the LSDs.

### Operation and governance

A key element of the program is close collaboration between interested stakeholders and partners, who share a common vision. Operations are centralized through the NGO program administrators (Direct Relief, California, USA, and Project Hope, Maryland, USA), who support the overall program management. Founded in 1948, Direct Relief has worked to equip HCPs in resource-poor communities around the world to meet the challenges of diagnosis and caring for people [10]. Direct Relief’s tradition of direct and targeted assistance over 70 years has evolved, and they have gained substantial expertise in the management of challenging logistics to identify charitable healthcare facilities and to deliver therapies into countries. These therapies must be delivered promptly, safely and in compliance with all temperature, storage and administrative regulations, with all arrangements being subject to rigorous quality control.

Project Hope is a global health and humanitarian relief organization with the aim of empowering HCPs to provide medical care to patients in need across the globe [11]. Since 1958, Project Hope has targeted healthcare emergencies and underserved communities in more than 100 countries, providing training to medical professionals and providing medicines and supplies [11]. Among its programs, Project Hope supports the logistics of ERT supply to 12 patients with GD in Egypt as part of the Takeda LSD charitable access program.

**Fig. 1:**
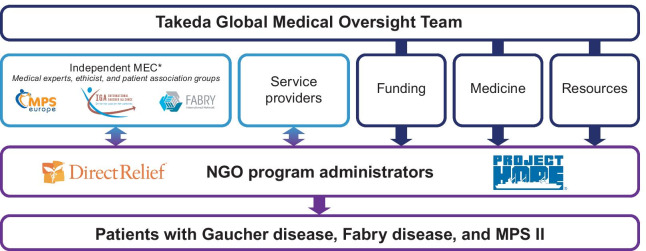
Overview of the current operating model of the LSD charitable access program. *LSD* lysosomal storage disorders, *MEC* medical expert committee, *NGO* non-governmental organization. *Direct Relief MEC. Project Hope is supported by a separate MEC

Direct Relief and Project Hope–managed programs are both supported by separate, independent medical expert committees (MECs), consisting of global LSD medical experts, representatives from rare disease patient organization groups, and an ethicist. All members of both program MECs volunteer their time. An overview of the Takeda LSD charitable access program operational model is shown in Fig. 1.

The Takeda charitable access program, which includes the MEC (supported by Direct Relief) and representatives from Direct Relief and Takeda, meet in person twice annually, or more frequently as required, for patient selection, to provide overall program strategy guidance and ongoing developmental oversight of program implementation. This includes selecting the countries and the approach to country capacity-building, selecting individual patients to receive treatment among those applying to the program, overseeing patient enrollment, and monitoring the response to treatment over time. MEC members from patient organizations provide critical advice on the most pressing needs in a given country as well as availability of existing in-country capabilities such as potential local program partners, physicians, and local patient representatives. Direct Relief and Takeda representatives and MEC members from patient organizations are non-active participants in individual case discussion and have no decision-making role in patient enrollment and monitoring.

There is a concerted effort to reach out to the local communities once enrollment periods are open, and outreach into the local patient and physician community is achieved in 2 main ways: (a) through the global patient organizations, which are members of the program, via direct request or through their own patient databases, and (b) by approaching appropriate physicians to invite them to attend educational sessions by way of introduction to the program. Through this latter approach, word of the program is passed through the communities of attending physicians.

### Patient selection

Criteria for patient selection are established by the independent group based on existing guidelines or expert consensus. These include clinical aspects such as presence of adequate diagnostic confirmation, physical and cognitive status, laboratory results, functional tests, imaging results, likely response to treatment and likely duration of treatment, as well as nonclinical aspects such as access to appropriate storage facilities, parental/guardian level of support, and assurance of weekly/twice-weekly access to a treating center with experience in handling infusions. Neither age nor gender are considerations for qualification to participate.

Applications for enrolment into the LSD charitable access program are submitted to Direct Relief, either directly by the patient’s physician, or through patient organizations. The application portal, which includes appropriate wording to provide consent, is shared by Direct Relief with submitting physicians. As part of the application, physicians have the option to upload a short video of their patient as relevant to the disease (e.g., eye movement for GD, gait for MPS ll). This provides greater clarity to the MEC of the patient’s clinical status and helps with the clinical assessment of the patient’s disease severity and to estimate the potential benefits of treatment. The medical experts independently review each application (with identifying details, i.e. the submitting HCP and country of applicant, removed) and provide their recommendation “YES—recommend enrollment”, “MAYBE (additional information needed)” or “NO—not suitable for enrollment” at MEC meetings. Each case is then jointly discussed by all medical experts at the face to face MEC meetings, reconciling any differences to reach consensus on which applicants to recommend for enrollment into the program. Input from the MEC medical ethicist into these discussions provides a very valuable perspective. The LSD medical experts on the MEC are the sole decision-making body for patient selection, with no involvement from Takeda. Once a patient is accepted for enrollment into the program, Direct Relief notifies the treating physician and ensures that the ERT drug supply can reach the approved treatment center efficiently, adhering to all local country regulations, and the highest global standards for drug supply logistics, including controlled temperature monitoring. For those patients who are not approved, information pertaining to further possible avenues for support is provided by the MEC to the applying physician. Physicians may consider re-applying at a later date should there be a significant change in the situation that justifies a reassessment.

### Patient enrollment into the LSD charitable access program

**Fig. 2:**
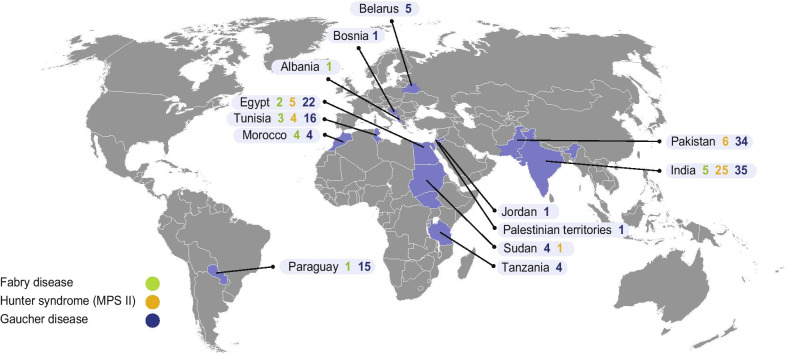
Patients enrolled into the LSD charitable access program, by country (*N* = 199). *LSD* lysosomal storage disorder

**Fig. 3:**
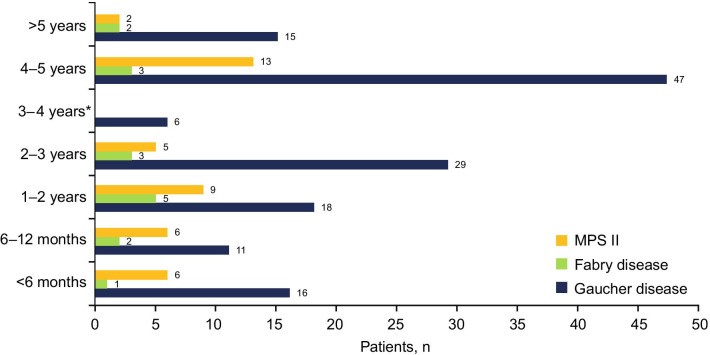
Duration of enrollment in the LSD charitable access program (*N* = 199). *LSD* lysosomal storage disorder, *MPS II* mucopolysaccharidosis type II (Hunter syndrome). *Few applications were made to the program in 2015, owing to limited remaining available places on the program and operation in countries with limited diagnostic facilities

As of August 31, 2019, 199 patients from 13 countries have been enrolled in the Takeda LSD charitable access program: 187 supported by Direct Relief and 12 by Project Hope. Countries with the greatest numbers of participants were India (*n* = 65), Pakistan (*n* = 40), Egypt (*n* = 29, including 12 patients from Project Hope), and Tunisia (*n* = 23) (Fig. 2). Of these, the majority (142 [71.4%]) were diagnosed with GD (107 [75.4%] with type I GD), 41 (20.6%) were diagnosed with MPS II, and 16 (8.0%) were diagnosed with FD (Table 1). The majority of patients were male (140 [70.4%]), which is unsurprising as 2 of the 3 conditions are inherited as X-linked traits. Overall, 95 (47.7%) patients were aged 0–10 years, 68 (71.6%) of whom were diagnosed with GD (Table 1). The length of time patients remain enrolled in the program is high, with 157 (78.9%) patients enrolled for at least 1 year, and 88 (44.2%) patients enrolled for ≥ 3 years (68 GD, 5 FD, and 15 MPS II; Fig. 3).

**Table 1:** Patient characteristics

	Gaucher disease	Fabry disease	Hunter syndrome (MPS II)
N	142	16	41
Sex, *n* (%)			
Male	84 (59.2)	16 (100.0)	41 (100.0)
Female	58 (40.8)	0	0
Age category, years			
0–10	68	0	27
11–20	34	0	12
21–30	14	9	2
31–40	9	4	0
41–50	10	2	0
> 51	7	1	0

## Results

### Patient monitoring and long-term follow-up

Once treatment is initiated, regular monitoring and follow-up are strongly recommended to ensure that patients are responding appropriately to treatment; for this reason, patients must receive treatment at the clinic as needed for infusions and undergo routine blood tests to continue in the program. Patient follow-up clinical data are reviewed at least annually by the MEC, while physicians are invited to contact clinical experts on the MEC for advice and support as needed.

**Fig. 4:**
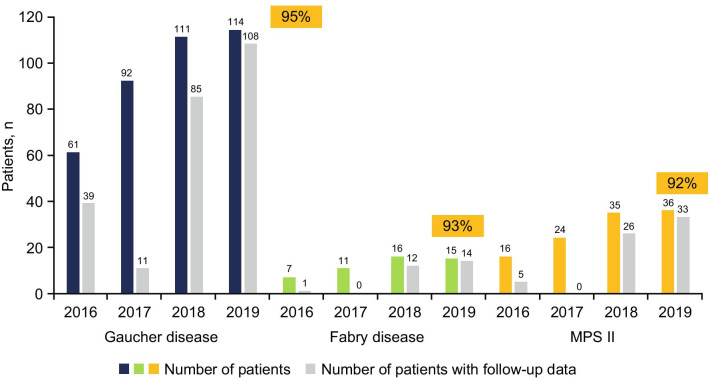
Follow-up reports at ≥ 12 months received per disease (2014–2019). *MPS II* mucopolysaccharidosis type II (Hunter syndrome). Excludes newly enrolled patients

**Fig. 5:**
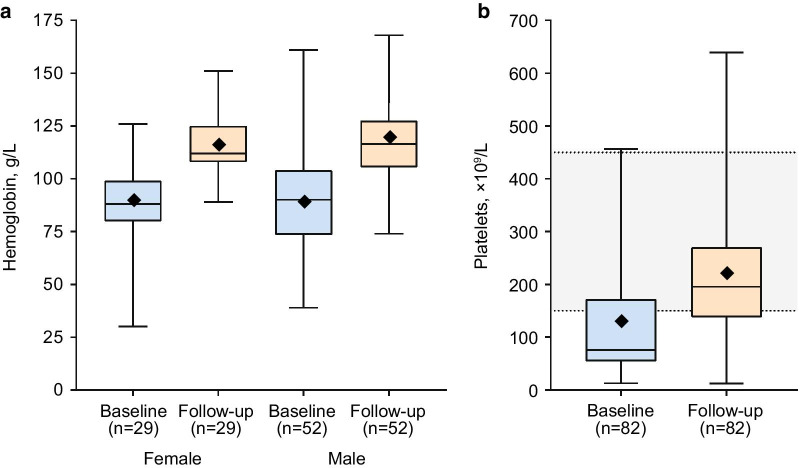
Change in hemoglobin levels (**a**) and platelet counts (**b**) in patients with Gaucher disease from baseline to follow-up at 1 year. Data shown are median, interquartile range, minimum, maximum values. Mean is indicated with (shaded diamond). Hemoglobin normal values: ≥ 116–164 g/L (females) and ≥ 127–181 g/L (males); platelet count normal range: 140–450 × 10^9^/L [21]

As of August 31, 2019, follow-up data were available for 155 of 165 (93.9%) patients for whom data were expected, all of whom were managed by Direct Relief; 108 with GD (90 with type 1 GD, 17 with type 3 GD, 1 with GD of unknown type), 14 with FD, and 33 with MPS II (Fig. 4). Of 108 patients with GD, 30 (27.8%; 24 with type 1 GD and 6 with type 3 GD) had undergone total splenectomy. Of these, 19 had total splenectomy prior to enrollment in the program, and 2 had splenectomy post-ERT initiation (unsupported by the program, which does not include general medical care); data were not available for 9 patients. Improvements in patients’ clinical condition over the previous year were reported by treating physicians for 147/155 (94.8%) patients with data, including 104/108 (96.3%) patients with GD, 12/14 (85.7%) patients with FD, and 31/33 (93.9%) patients with MPS II. Improvements in hematologic parameters were achieved in most patients with GD. Of 81 patients with GD with available data, median (range) hemoglobin levels increased from 90 (30–161) g/L at baseline, where values < 110 g/L (females and children ≤ 12 years) or < 120 g/L (males) are suggestive of anemia, to 115 (74–168) g/L at follow-up, a median increase of 27 (− 16 to 94) g/L, or 27.9%. Similar increases were observed in males (*n* = 52) and females (*n* = 29) (Fig. 5; Table 2). In 82 patients with GD, median (range) platelet count increased from 76.0 (13.0–456.0) × 10^9^/L at baseline, where values < 150 × 10^9^/L are suggestive of thrombocytopenia, to 195.5 (12.0–639.0) × 10^9^/L at follow-up, a median increase of 72.0 (− 155.0 to 540.0) × 10^9^/L, or 82.4% (Fig. 5; Table 2).

**Table 2:** Change in disease parameters from baseline to follow-up in patients with Gaucher disease

	Baseline	Follow-up	Change
Hemoglobin, g/L			
Total (*n* = 81)			
Mean (SD)	89 (22.1)	117 (19.7)	28 (20.5)
Median (range)	90 (30–161)	115 (74–168)	27 (− 16 to 94)
Male (*n* = 52)			
Mean (SD)	89 (23.0)	119 (21.9)	30 (21.7)
Median (range)	90 (39–161)	117 (74–168)	30 (− 16 to 94)
Female (*n* = 29)			
Mean (SD)	89 (20.8)	115 (15.2)	26 (18.3)
Median (range)	88 (30–126)	112 (89–151)	21 (1–79)
Platelet count × 10^9^/L			
Total (*n* = 82)			
Mean (SD)	127.14 (109.56)	217.88 (125.83)	90.74 (120.27)
Median (range)	76.0 (13.0–456.0)	195.50 (12.0–639.0)	72.0 (− 155.0 to 540.0)

*SD* standard deviation

Physicians reported no improvements in the condition of 4 (3.7%) patients with GD: 1 patient with type 3 GD had progression of neurological features, 1 patient with type 3 GD had increased spleen size, 1 patient with type 1 GD had Parkinson’s disease that limited improvements in quality of life, and 1 patient with type 3 GD had increased hepatosplenomegaly. No improvement was reported for 2 (14.3%) patients with FD and in 2 (6.1%) patients with MPS II. Of these, 1 FD patient is on renal dialysis, whereas the other with no significant clinical improvement had weight loss and pain in both hands and feet; 1 MPS II patient had disease progression, becoming non-ambulant, and the other had no change in joint stiffness and development of significant infusion-related reactions.

### Discontinuations

Although Takeda has committed to a life-long drug supply for each supported patient, treatment through the program may be stopped at the discretion of the independent medical experts on the MEC for the following reasons: if the patient is deemed noncompliant with treatment, shows no response to treatment, develops another life-threatening condition, develops a serious adverse reaction, or if no patient-follow-up information is provided within the specified period. Every effort is made to explore options to facilitate compliance before discontinuing ERT, and decisions are made in agreement with the local treating clinician on a case-by-case basis. Patients, families, or their physicians may also choose to discontinue treatment at any time for any reason. A patient information leaflet is provided to all patients before enrolling in the program, which includes an overview of each disease covered by the program, and answers to frequently asked questions. Since the beginning of the program (2014), a total of 37 patients dropped out or discontinued; 10 were enrolled but did not receive treatment, 9 discontinued treatment for poor compliance (MEC expert decision, *n* = 6, or locally treating HCP decision, *n* = 3), 7 patients switched to another ERT product that had become available locally, and 11 patients died, 6 with Gaucher disease, and 5 with MPS II. Of the patients who have passed away during this period, 5 patients were enrolled in the program for less than 2 years, and 6 patients were enrolled between 2 and 6 years.

### Capacity building

Local physicians with patients enrolled in the program receive ongoing, long-term support and appropriate education in collaboration with the program’s partners to ensure best practice for ongoing administration of treatment. These activities include a program of educational events organized and delivered by NGO partners, with content created by experts and committee members, supported by an independent educational grant from Takeda. Over the past few years, such independent educational events have been held in several countries, including Morocco, Kazakhstan, Panama and Botswana, to which interested physicians from the regions and countries surrounding the event are invited to attend. Such educational events have successfully provided information and training to local physicians on LSDs as well as other rare inherited metabolic disorders. The topics cover support with diagnosis, non-drug management options, surgical management, pregnancy management, concomitant medications for joint pain, psychological input, etc., and management of complications including the prevention and management of infusion-related reactions. Post-event online learning modules provide ongoing training for event attendees; the materials focus on reinforcing rare disease awareness and diagnostic support. Thirty physicians are currently registered on the associated post-event online learning platform, working through a total of 30 learning modules [12]. Follow-up educational events, organized by FYMCA Medical Ltd, independent of Takeda, are also held for those who have completed online training modules.

Additionally, small-group clinical “hands-on” preceptorship-type meetings are organized at centers with many years of experience in management of LSDs, led by a faculty including members of the MEC, and supported by independent educational grants from Takeda. These are open to HCPs with patients enrolled in the program (travel supported by Takeda). These meetings provide local physicians who have patients actively enrolled in the program with hands-on education for disease recognition; definitive diagnosis and management of their patients with FD, GD or MPS II; and the opportunity to network and share experiences with other physicians facing similar local challenges. Delegates also have the opportunity to present challenging patient cases and request advice from the faculty. After these meetings, physicians actively participate in local dissemination of knowledge and expertise among their local teams, thereby continuing the education locally to include all those managing these LSD patients. For some of the clinicians, these preceptorship meetings might be the only training support and networking opportunities they receive regarding LSDs and hence serve an important, integral part of the charitable access program.

## Discussion

Working collaboratively with several dedicated stakeholders, the Takeda LSD charitable access program has provided a holistic approach to patient care, in part by providing stable and continuous access to ERT for patients in underserved communities. Since its inception, 236 patients across 13 countries have been enrolled into the program, with 199 patients remaining active in the program as of August 31, 2019. Of these, a considerable proportion have been enrolled for ≥ 3 years, demonstrating the long-term local sustainability of the program.

Overall, there has been an increased enrollment of male patients in the program, compared to females, at an approximate ratio of 7:3 males to females. This can be explained in part by the fact that MPS II, as an X-linked disorder, affects almost exclusively males, and although FD may affect more females than males, severe symptoms may occur earlier and more frequently in males than in females [13]. Indeed, all FD patients enrolled in the Takeda LSD charitable access program were male. For GD patients, the proportions of males and females are 59% and 41%, respectively. These gender disparities exist despite the program design, with applications considered equally during the selection process, regardless of gender. The majority of patients enrolled in the program have a diagnosis of GD, whereas only 16 have a diagnosis of FD. This low number of FD patients is not consistent with the published worldwide FD epidemiological data. Explanation for the low numbers of FD patients enrolled is not clear but may be the result of a combination of factors leading to FD not being diagnosed or even mis-diagnosed in a local country. Contributing factors could include a lack of local disease awareness, lack of access to diagnostic facilities, and the complexity of diagnosing FD, which has a wide range of initial clinical presentations even in patients with the same genetic mutation. GD and MPS II often show a typical set of clinical features in children, which facilitates an understanding of disease awareness and perhaps a clearer path to diagnosis, compared with FD. Lack of local specialist expertise and supportive services, including the biochemical and genetic tests required for a definite diagnosis, present hurdles to improving the rates of detection of FD in underserved countries. Increased FD awareness and accurate diagnosis are urgently needed to support undiagnosed and misdiagnosed patients, and educational awareness programs to aid diagnosis are a core part of the LSD CAP program. This could be achieved through continued efforts to raise disease awareness, diagnostic support, infrastructure development, local public health prioritization, and access to treatment.

Nearly a third of patients with GD in the program had undergone total splenectomy prior to enrollment. Splenectomy was considered standard of care for GD prior to the introduction of ERT in 1991, with ~ 43% of the adult GD population undergoing the procedure [14]. This still remains standard of care in some regions with no access to pharmacological therapy, where management has changed little since reports from the 1970s and 1980s [15–17]. However, in established health care systems, total splenectomy is rarely performed in GD patients being treated, and is considered only for patients with massive splenomegaly and severe hypersplenism *despite* treatment with ERT [14, 18]. The low number of patients undergoing splenectomy after enrolling in this program supports the supposition that availability of ERT reduces the need for total splenectomy in patients with GD.

Our follow-up data indicate improvements in patients’ clinical condition for a large majority of patients. Although many programs do not collect or report outcomes data from patients in receipt of donated medicines, annual follow-up is strongly recommended in the Takeda LSD charitable access program to ensure that patients continue to benefit from on-going treatment and to highlight areas of the program in need of improvement. The physician response rate for patient follow-up as part of the program was high, with data collected for more than 90% patients enrolled in the program for > 1 year. This is despite challenges faced in certain countries/regions in the collection of outcomes data, e.g., remote locations, patient difficulty in accessing healthcare centers, lack of laboratory facilities for monitoring disease parameters, and increased workload associated with the effort. However, a small proportion of patients and/or physicians have difficulty in meeting the requirement of reporting annual outcomes data. Missing follow-up data do not automatically result in discontinuation from the program but rather trigger attempts to help physicians and patients to comply with data requirements. A continued focus on obtaining this information is paramount, particularly in remote regions and charitable-type programs.

Although data on long-term outcomes of patients enrolled in the charitable access program are limited, a small subset of GD patients enrolled in the program had previously participated in velaglucerase alfa clinical trials and have additionally enrolled into the Shire/Takeda Gaucher Outcome Survey, an international global patient registry, operational in countries in which the product is commercially available [19]. This enables the collection of more detailed longitudinal outcomes data for patients receiving velaglucerase alfa through the program. It is expected that more patients from the program will enter the Gaucher Outcome Survey as more sites participate in the registry.

A limitation of the analyses for these results is incomplete data availability for disease characteristics. It is important to also understand the situation that physicians in some of these countries are typically covering a huge population with very limited resources. Therefore, the “demand” for data needs to be always weighted against the feasibility and burden this puts on patients, families, and physicians. Furthermore, owing to the need for data protection and patient confidentiality, detailed evaluation of country-level data cannot be supported due to the small numbers of patients per individual country. However, it is essential—to the extent possible and appropriate—to have longitudinal safety and effectiveness clinical data in follow-up of patients on ERT. This is critical not only for monitoring long-term treatment and safety effects, but also to better understand the emerging history of (treated) patients in countries with otherwise very sparse—if any—efficacy and safety information for these types of diseases. The need for collection of follow-up data from patients on charitable access programs should be no different for patients with reimbursed treatments.

## Conclusions

Since inception of the program, methods of patient selection, enrollment and monitoring have evolved and will continue to evolve, based on learnings from the program itself. Nevertheless, the Takeda LSD charitable access program can help physicians, caretakers, and patients in underserved communities. More work and partnerships are needed to address the challenges and unmet needs of patients with LSDs and other rare diseases from these developing countries around the world. An important step toward this goal was achieved by the rare disease community on September 23, 2019 in New York at a high-level meeting at the United Nations (UN) headquarters, at which UN Member States adopted a political declaration on universal health coverage that includes mention of rare diseases [20]. This marks a significant milestone for the rare disease community, with rare diseases being included for the first time within a UN declaration adopted by all 193 Member States. Our early experience with this LSD charitable access program suggests that such programs can make a positive impact, allowing clinicians to help patients who previously would have had few or no options available. Although significantly more work is needed, this program marks one small step of many needed to accomplish the goal of “leave no one [with rare diseases] behind”.

## Data Availability

Study data are not publicly available for patient privacy reasons. Anonymized data can be made available upon reasonable request.

## Abbreviations

ERT: Enzyme replacement therapy
FD: Fabry disease
GD: Gaucher disease
HCP: Healthcare provider
LSD: Lysosomal storage disorder
MEC: Medical expert committee
MPS II: Mucopolysaccharidosis type II
NGO: Non-governmental organization
PO: Patient organization
SRT: Substrate reduction therapy
UN: United Nations
